# Neuroscience of Behavior

**DOI:** 10.3390/neurosci6040108

**Published:** 2025-10-24

**Authors:** Mario Treviño, Oscar Arias-Carrión, Braniff de la Torre-Valdovinos, Paulina Osuna Carrasco, Inmaculada Márquez

**Affiliations:** 1 Laboratorio de Plasticidad Cortical y Aprendizaje Perceptual, Instituto de Neurociencias, Universidad de Guadalajara, Guadalajara 44130, Mexico; 2 División de Neurociencias, Clínica, Instituto Nacional de Rehabilitación Luis Guillermo Ibarra Ibarra, Mexico City 14389, Mexico; 3 Tecnologico de Monterrey, Escuela de Medicina y Ciencias de la Salud, Mexico City 14380, Mexico; 4 Laboratorio de Neurofisiología, Departamento de Bioingeniería Traslacional, Centro Universitario de Ciencias Exactas e Ingenierías (CUCEI), Universidad de Guadalajara, Guadalajara 44430, Mexico

**Keywords:** behavioral neuroscience, neural dynamics, systems integration, latent variables, action planning

## Abstract

Behavior is not a mere sequence of responses to stimuli but the dynamic expression of internal processes such as planning, prediction, valuation, and inference. These functions arise from distributed and metabolically costly neural systems and are best understood by considering behavior and neural activity together. This article presents a narrative and conceptual review of the neuroscience of behavior, integrating biological, environmental, and computational perspectives. We synthesize evidence from motor control, neural population dynamics, predictive processing, and spontaneous behavior, showing that behavior cannot be explained without the neural systems that generate it, and that neural activity gains meaning only through detailed behavioral models. Neural dynamics correlate with latent variables, such as intention and prediction error, that structure adaptive action across timescales. Recent advances in behavioral analysis, dimensionality reduction, and computational modeling enable the analysis of neural and behavioral data with comparable complexity, revealing shared computational architectures that link population activity with the organization of action. Our methodology involved a targeted literature search in PubMed and Web of Science (1919–2025), supplemented by seminal earlier works. By combining mechanistic and functional analysis, we outline a unified framework that explains how brains, bodies, and environments together generate flexible, adaptive behavior.

## 1. Introduction

Scientific disciplines evolve through methodological advances, conceptual shifts, and broader explanatory frameworks. Behavioral science and neuroscience have each contributed essential perspectives: the former focusing on observable contingencies, structured environments, and learning histories; the latter on the organization and dynamics of nervous systems. Together, these approaches offer complementary accounts of the same phenomenon: behavior as a biological process shaped by neural dynamics, environmental contexts, and adaptive demands.

Current evidence highlights the importance of this integration. Systems neuroscience shows that behavior arises from distributed neural processes spanning spinal circuits, cortical hierarchies, and subcortical loops, supporting prediction, action selection, working memory, and valuation [1,2]. At the same time, behavioral research makes clear that neural activity can only be fully understood within the environmental conditions, histories, and goals in which organisms act. Viewed jointly, behavior and neural activity emerge as interdependent elements of a unified control architecture.

The aim of this article is to examine how combining behavioral science and systems neuroscience can deepen our understanding of behavior as a biological phenomenon. We advocate for a conceptual synthesis that integrates mechanistic explanations of neural activity with functional analyses of adaptive behavior.

The objectives of this review are threefold: (i) to provide a conceptual synthesis linking neural, behavioral, and environmental levels of explanation; (ii) to outline methodological and computational tools that enable this integration; and (iii) to identify potential future directions, including experimental designs and clinical applications. This is a narrative and conceptual review, based on a targeted literature search in PubMed and Web of Science (1919–2025), complemented by earlier seminal works. Inclusion criteria prioritized studies that provided mechanistic insights into neural control of behavior, theoretical and computational models bridging neural and behavioral domains, and conceptual contributions proposing integrative frameworks. By treating behavioral paradigms and systems neuroscience as mutually informative, we aim to contribute to a more comprehensive account of adaptive action: one that explains how brains, bodies, and environments together generate flexible behavior.

## 2. Structured Complexity of Behavior

Behavior is a dynamic phenomenon shaped by the interplay of internal states, environmental contexts, and learning histories [3,4,5]. Internal states like intention, motivation, and arousal structure action across multiple timescales. High-resolution tracking confirms that continuous action is temporally and spatially structured. For example, in mice, three-dimensional motion capture with unsupervised learning decomposes behavior into sub-second “syllables” that follow probabilistic sequences rather than random patterns [6]. In mice, some transitions follow Markovian rules, where the next action depends only on the current state, while others are non-Markovian, shaped by prior history and internal context. Invertebrates such as Drosophila display non-Markovian locomotor submodes shaped by recent history [7], while zebrafish decisions reveal long-range dependencies modulated by physiological state, such as hunger [8]. Spontaneous behaviors like grooming or exploration in mice also exhibit coherent structure and vulnerability to perturbation: cerebellar or striatal manipulations systematically alter their microstructure, linking neural state to behavioral organization [9].

Computational approaches converge on the view that behavior reflects low-dimensional latent states shaped by neural dynamics and environmental feedback [10]. Machine learning extends this principle by segmenting rodent behavior into modules governed by probabilistic rules, linking micro-actions to cognitive goals without the necessity of employing ‘human labels’ [6,11]. Hierarchical decomposition scales across species and timescales, from millisecond steps to extended rituals such as courtship or speech, revealing conserved motifs that expose gene–behavior links. These findings resonate with long-standing conceptual frameworks. Aristotle distinguished efficient, formal, material, and final causes; Marr articulated computational, algorithmic, and implementation levels; and Tinbergen emphasized mechanism, function, ontogeny, and phylogeny. Each stresses that behavior cannot be reduced to a single level of explanation. Instead, comprehensive accounts require integration across triggers, representations, substrates, adaptive goals, developmental histories, and evolutionary contexts [9,11,12].

Neural population dynamics mirror this structured complexity. Population activity unfolds along low-dimensional trajectories that capture both motor kinematics and internal states [13]. Even noisy single-trial traces follow consistent paths, revealing modular and hierarchical organization across timescales. Just as behavior is composed of syllables, sequences, and goals, neural activity is organized into states and trajectories. This parallel organization reveals the inseparability of behavioral structure and neural dynamics in explaining adaptive action.

## 3. A Neural Basis of Motor Control

Voluntary movement is the most direct and measurable expression of behavior, generated through distributed neural circuits spanning the spinal cord, brainstem, cortex, and cerebellum. These circuits not only permit movement but also generate, coordinate, and adapt it through hierarchical control loops. Evidence derives both from experiments linking specific neural activity patterns to discrete outputs and from disorders in which their disruption impairs function.

At the foundational level, the spinal cord contains genetically defined microcircuits that coordinate basic ‘motor primitives’. Central pattern generators (CPGs) produce rhythmic locomotor patterns such as walking, swimming, or scratching even without supraspinal input. Their modular design allows autonomous operation while remaining sensitive to descending commands [14]. The brainstem issues command-like signals for initiating actions. Thus, for example, stimulation of specific nuclei elicits stereotyped behaviors such as locomotion, freezing, or orienting. Inputs from basal ganglia, cortex, and sensory systems provide contextual and motivational modulation, and lesions or optogenetic silencing here produce deficits in initiation or regulation [14].

The motor cortex functions as a dynamic generator of action plans rather than a simple trigger. During preparation and execution, population activity evolves along smooth trajectories that encode the direction, speed, and timing of movement [15,16,17]. These trajectories reflect structured pathways in high-dimensional activity space, separating preparatory activity from motor output. Here, synaptic inhibition further reorganizes cortical activity, decorrelating nearby neurons to enhance information-processing efficiency [18]. Perturbations of motor or even somatosensory cortex impair execution and coordination, illustrating their feedforward role in control [19].

The basal ganglia and cerebellum provide complementary layers of control. Dopaminergic neurons in the substantia nigra pars compacta regulate timing and initiation through movement-related pauses; their loss in Parkinsonian models alters striatal dynamics before overt cell death [20]. The cerebellum refines precision and coordination via Purkinje cell modulation of deep nuclei. Transient inhibition of Purkinje cells evokes rapid, scalable movements with microzone-specific effects, showing that cerebellar circuits encode executable commands rather than serving only corrective feedback [21].

Motor control also reflects interactions between neural circuits, body morphology, and ecological context. Cortico-motoneuronal connections in primates support precision grips by matching spinal outputs to hand biomechanics. In humans, infants stop stepping not because the brain suppresses it, but because their legs become too heavy; the behavior returns when the body is supported and the weight is reduced (for example, in water). Similarly, ferret ear growth recalibrates auditory spatial encoding without requiring immature circuits to relearn [22]. Naturalistic tasks further illustrate these principles: during prey capture, rodents coordinate bilateral eye and head movements, alternating compensatory and non-compensatory strategies. Visual cortex transforms incomplete cues into accurate motor outputs, highlighting the ecological embedding of motor control [11].

Advanced modeling reinforces a dynamical view. Single-trial spiking reveals motor cortical activity evolving predictably from trial-specific initial conditions, enabling high-accuracy decoding of hand trajectories and reframing control as trajectory shaping within neural state space [13,23]. ‘Behavioral primitives’, from saccades to navigation, are organized hierarchically across species. In Drosophila, grooming follows suppression hierarchies; in songbirds, basal ganglia disruptions impair vocal plasticity. Automated tracking and clustering link such primitives to neural circuits, yielding mechanistic models of control [10].

Across these levels, the primary motor cortex and associated circuits orchestrate a diverse repertoire through stable yet flexible population structures. Covariance patterns remain consistent across tasks, supporting motor adaptability without fixed neuron-to-parameter mappings [17]. This stability provides a robust substrate for skilled behavior, from tool use to social interaction, revealing the principle that adaptive motor control emerges from coordinated, hierarchical, and dynamically structured neural population activity.

## 4. Population Dynamics and the Manifold Structure of Neural Control

Understanding behavior requires more than identifying which neurons are active during an action, it demands a mechanistic account of how coordinated population activity evolves to generate, constrain, and adapt movement. Neural activity during planning, execution, and decision-making is structured and constrained, occupying low-dimensional subspaces, or neural manifolds, that capture the dominant modes of network activity. These manifolds emerge from network-level connectivity and plasticity rather than the tuning of individual neurons, and a small number of modes often explains most of the variance in population activity [16,24].

Within motor cortex, activity unfolds along temporally organized trajectories in these manifolds, encoding parameters such as movement direction, force, and timing [16,25]. This organization separates null spaces, which support preparatory activity without influencing motor output, from potent spaces that project to effectors and directly drive movement [17,23]. Such separation enables the brain to plan flexibly while preventing premature execution. Similar low-dimensional population structures govern hierarchical control in biological systems as diverse as C. elegans, where manifold trajectories encode transitions between high-level behavioral states and fine-grained motor patterns, effectively capturing the ‘grammar’ of behavioral sequences [25,26]. Moreover, learning interacts with manifold geometry: adaptation proceeds rapidly when tasks can be solved by modifying existing trajectories, but is slower when novel dynamics outside the intrinsic manifold must be established [27,28]. This property constrains future behavior and provides a foundation for generalization across related tasks. Manifolds appear across species and cortical areas, including associative, prefrontal, and hippocampal networks, where they encode decision variables, memory states, and navigational goals [1,29]. In rodent hippocampus, for example, manifold trajectories branch before overt choices, predicting future actions up to a second in advance, and shift from spatial to goal-related encoding as a decision point approaches [29].

Manifolds can also represent movement-irrelevant but behaviorally critical information. During uncertain decision-making, multiple potential actions are ‘simulated’ in parallel as competing trajectories within the manifold [30,31]. Frontal and cerebellar regions show ramping activity that encodes expected outcomes, while gain modulation suppresses competing plans to prevent premature execution [32,33,34]. Importantly, even without overt movement, such preparatory dynamics are energetically costly and distributed across cortical and subcortical structures.

Advanced modeling techniques, including principal component analysis (PCA), Gaussian process factor analysis (GPFA), and switching linear dynamical systems (SLDS), can capture these dynamics by aligning unmeasured internal states with behaviorally relevant variance [13,35,36]. This framework parallels computational behavioral representations, such as in C. elegans or fly postural modes, where complex kinematics reduce to low-dimensional trajectories that reconstruct original behaviors with high fidelity [10]. Therefore, these behavioral manifolds, like their neural counterparts, reveal how structured variability emerges from constrained trajectories.

The manifold framework extends to specific motor systems. In motor cortex, rotational dynamics, once thought to emerge only in trial-averaged data, persist in single trials, revealing oscillatory patterns in primary motor cortex (M1) and dorsal premotor cortex (PMd) that generalize to novel movement conditions [13]. Preparatory activity aligns more closely with a perimovement space, defined by principal population patterns, than with traditional kinematic task spaces, illustrating how motor control depends on collective dynamics rather than isolated neuron tuning [23]. In the cerebellum, spatially localized Purkinje cell inhibition produces proportional changes in deep cerebellar nucleus firing and movement kinematics, suggesting that cerebellar output also operates within a manifold where inhibitory population dynamics map onto precise motor parameters [21].

Across motor tasks, M1 maintains stable covariance structures, shared neural modes, despite changes in behavioral demands. These modes, which account for much of the variance in neural activity, flexibly combine to generate task-specific motor commands [17]. Similarly, in navigational and orienting systems, population activity forms continuous attractor manifolds, such as the one-dimensional ring in the Drosophila head direction system or the two-dimensional torus in mammalian grid cells. These geometries persist across contexts and behavioral states, reflecting intrinsic circuit connectivity that supports stable, high-fidelity representations of spatial variables [26].

## 5. Organisms Operate Under Incomplete Information

Organisms do not perceive a complete or stable representation of the world. Indeed, environments are noisy, ambiguous, and partially observable; sensory input is often degraded, occluded, or missing. Yet behavior remains robust and purposeful. One possibility is that this capacity arises from internal models that predict, simulate, and interpret sensory input, filling informational gaps and disambiguating signals. Predictive processing theory formalizes this principle: neural circuits encode both expected inputs and prediction errors, with recurrent interactions continuously updating internal models to minimize future surprise [37,38,39]. These models evolve with learning and context, sustaining useful representations even when direct evidence is absent.

Predictive processing reframes behavior as prospective. Thus, animals do not merely react; they anticipate outcomes, evaluate options, and act based on expectations. Behavioral variability often reflects attempts to resolve ambiguity rather than noise, a fact that constrained laboratory paradigms may obscure [10]. Moreover, even randomized designs can induce biases: subtle temporal or spatial regularities in “chance” sequences drive systematic choice tendencies [40]. Decision criteria are further shaped by error-correction heuristics, producing systematic deviations from optimality under low discriminability or uncertain contingencies [41]. Natural behaviors highlight all these principles. In navigation, mice integrate ambiguous local cues over time, forming probabilistic representations of position and selecting paths despite uncertainty. Retrosplenial cortex activity reflects “uncertain” states, with intermediate, hypothesis-updating dynamics resembling foraging choices under ecological constraints [11]. Associative learning likewise involves probabilistic updating: conditional probabilities quantify uncertainty reduction, with priors accelerating or slowing learning rates. Sparse prior experience enables rapid updating, as in taste aversion, while extensive exposure slows change, with contextual cues serving as external memory [12].

Circuit-level mechanisms also contribute to uncertainty processing. Neuromodulators such as norepinephrine dynamically adjust perceptual discrimination via divisive gain control in the cortex, reducing accuracy and increasing uncertainty [42,43]. Each species has its own way of filtering sensory information: for example, colored leg bands change mate choice in zebra finches, and looming sounds trigger earlier escape in weaker individuals, showing how perception adapts to body capabilities and ecological demands [22].

Neural dynamics suggest how predictions compensate for missing inputs. In auditory-guided navigation, mice infer target locations from truncated tone sweeps, with hippocampal activity ramping toward goals even without ongoing feedback. Neural trajectories align with chosen paths, with stronger activation preceding unlikely choices and diminishing as uncertainty resolves [29]. In motor control, adaptive corrections to perturbations such as cursor jumps rely on cortical populations integrating unmeasured inputs. Dynamical systems modeling reveals the timing and clustering of these hidden influences, showing how circuits can participate in inferring environmental states to guide behavioral responses [13].

## 6. Movement-Irrelevant Neuronal Activity and the Internal Architecture of Behavior

Not all neuronal activity leads directly to overt movement. A substantial portion of brain dynamics supports internal computations, planning, decision-making, action selection, valuation, and goal maintenance, that occur before or without observable behavior. These processes are essential for adaptive control and rely on distinct, distributed neural circuits. In the motor system, preparatory activity precedes execution by hundreds of milliseconds and evolves within a null space of population activity, dimensions that do not project onto muscle output. The premise is that this subspace allows flexible specification of action plans while preventing premature initiation [16,23,33]. Motor cortex, cerebellum, and frontal cortex display structured trajectories during this phase, encoding and maintaining intended actions [32], while prefrontal and parietal areas sustain task-relevant variables during delayed responses, enabling temporary storage and flexible recall of goals [1]. In the hippocampus, neurons are thought to encode internal states such as intended trajectories or uncertainty about goal location, integrating contextual and past information to update action policies [29]. Across cortical and subcortical regions, movement-irrelevant signals often ramp in parallel for multiple competing action plans, producing transient ‘bottlenecks’ in rapid or sequential decision-making tasks [30,31,44].

Adaptive behavior requires not only the activation of selected motor representations but also the suppression of competing outputs. During preparation, task-irrelevant circuits are inhibited through additive suppression and gain modulation, enhancing contrast between alternatives and ensuring accurate execution [34]. These dynamics integrate past experience with current environmental demands, relying on stable trajectories over time and coordination between distributed systems. The cerebellum sustains frontal cortex preparatory activity, ensuring temporal precision [33]. Even early sensory cortices contribute to this internal framework: variability in sensory responses reflects multiplicative modulation along stimulus-encoding dimensions and additive contributions from behavior-related activity, such as arousal, posture, or facial movements [2,45]. Behavioral and sensory representations often occupy orthogonal population subspaces, allowing stimulus-specific signals to be integrated without disrupting ongoing internal computations. A single shared dimension tracks global state fluctuations, while orthogonal structures preserve parallel encoding of internal and external variables [46]. This ‘multiplexed’ organization indicates that spontaneous activity is not mere replay of sensory input but an active encoding of behavioral state. In naturalistic conditions, sensory processing is dynamically configured by locomotion, expectation, engagement, and reward history, emerging from recurrent interactions across cortical and subcortical circuits [47]. Thus, behavior is the endpoint of a dynamic internal architecture in which movement-irrelevant activity provides the substrate for anticipation, planning, suppression, and adaptive context setting. Even in constrained tasks, movement-irrelevant components shape cortical activity. For example, in head-fixed rodents performing decision tasks, small “spurious” movements, once dismissed as noise, account for significant neural variance and help disambiguate sensory and memory-related activity. In freely moving animals, such as during social interactions, postural dynamics in the dorsal medial prefrontal cortex change with the presence of conspecifics, encoding internal states such as social rank or motivation. Wireless recordings reveal that behavior emerges from the interplay of motor-relevant and non-motor neural manifolds, challenging reductionist accounts of action generation [11].

Movement-irrelevant activity also supports memory-based control. In conditioning, flexible responses initially depend on conscious processing but become automatized with practice, reflecting a shift from computation-intensive to more reflexive states. This transition relies on systems with limited memory, whether stored in the brain or marked in the environment, that slow down information loss and keep detailed histories available for later use. Such architectures enable a balance between adaptability and efficiency, with conscious processes permitting flexible adaptation and unconscious processes enabling rapid execution [12].

Decoupling from immediate movements can serve perceptual constancy and adaptability. Rats performing evasive maneuvers maintain distance from threats through varied motor patterns rather than fixed trajectories. In Drosophila, locomotor handedness shows heritable variability, suggesting that built-in stochasticity in neural control is a functional feature for coping with unpredictability [22]. Hippocampal spiking often reflects prospective sequences rather than current sensory input, ramping toward goals independently of reward delivery or spatial coordinates. During deliberative pauses, transient activation of goal-specific assemblies simulates possible outcomes without committing to action, and even in error or exploratory trials, firing patterns track intended rather than actual progression [29].

Modeling neural populations as dynamical systems reveals unmeasured factors that capture preparatory and contextual signals not seen in overt movement. In reaching tasks, these factors encode trial-specific initial conditions and unmeasured external inputs, such as target position before a perturbation. In cursor jump experiments, inferred inputs cluster by perturbation type and timing, showing that neural dynamics integrate contextual cues beyond immediate motor output [13]. Preparatory activity is often tuned for multiple parameters but lacks a consistent dominant factor, and its weak correlation with perimovement tuning suggests that it establishes the initial conditions for dynamical evolution rather than directly encoding movement parameters [23]. Time-related neural modes in M1, consistent across tasks and targets, further illustrate this point: they likely reflect intrinsic transitions from planning to execution, maintaining a reservoir of movement-irrelevant activity that stabilizes computations and facilitates behavioral flexibility [17].

## 7. A Broader Neurobiological Basis Supporting Behavior

Behavior arises from the coordinated activity of neurons embedded within a larger biological framework that includes astrocytes, microglia, vascular systems, metabolic dynamics, and the embodied structure of the organism. These elements actively support and constrain neural computations, enabling both real-time control and long-term plasticity. Astrocytes regulate synaptic transmission, metabolic supply, and calcium signaling, stabilizing network states and tuning contextual sensitivity [48]. Microglia contribute by pruning synapses and adjusting efficacy in response to experience, with dysfunction impairing flexibility and motivation. Astrocytic networks also mediate neurovascular coupling, ensuring that local blood flow and glucose delivery scale with task demands.

This foundation extends beyond isolated circuits to integrate sensory, motivational, and social processes across brain regions. In kinship recognition, for example, the lateral septum encodes affiliative versus avoidant responses in pups, while in social foraging, the striatum and prefrontal cortex coordinate rapid motor adjustments and postural transitions. Such patterns reflect an evolutionary architecture tuned for survival strategies such as generalist foraging [11]. Similarly, vocal communication in birds and rodents depends on feedback loops between acoustic features and neural circuits, with basal ganglia perturbations altering plasticity and wild contexts restoring natural social dynamics. Automated tracking now links genetic and neural manipulations to behavioral primitives across species, from insect courtship to rodent exploration, revealing how biological substrates scaffold adaptive repertoires [10]. Multiple explanatory levels illustrate this complexity. Neural circuits provide the physical substrate, but behavior also reflects efficient causes (stimuli and contingencies), formal causes (computational models of organization), and final causes (adaptive functions). Marr’s hierarchy of computational, algorithmic, and implementational levels complements this view by emphasizing problem-solving, representational rules, and physical instantiation. Tinbergen’s questions (mechanism, function, ontogeny, and phylogeny) further integrate proximate and ultimate dimensions. Together, these frameworks highlight that full explanations must bridge mechanisms, representations, development, and evolution, rather than privileging one level in isolation. Embodiment further shapes behavior. Morphological features filter sensory inputs and constrain motor outputs, as in how outer ear morphology calibrates auditory localization. Developmental milestones such as babbling co-occurring with sitting, or evolutionary adaptations like lactose tolerance, illustrate how brains, bodies, and environments co-constitute behavior [22]. Agency transforms reactive loops into proactive control, embedding action within individual history and species evolution.

At the systems level, shared population dynamics unify behavior across tasks and timescales. In the hippocampus, neural tuning to space, time, and rewards links sensory inputs to voluntary action sequences, sustaining memory, prediction, and planning in coordination with frontal areas [29]. Methods for aligning neural data show that distributed populations share common low-dimensional trajectories that remain stable over months, supporting generalizable control [13]. Inhibitory circuits add further precision: Purkinje cell suppression initiates and refines movements, with distinct cerebellar microzones driving specific motor acts [21]. Across cortical and subcortical networks, task-independent modes preserve manifold orientation, enabling new skills without overwriting old ones [17]. Finally, the architecture of recurrent circuits gives rise to low-dimensional manifolds that organize behavior. In flies, ring attractors encode heading direction; in mammals, grid-cell dynamics may form toroidal maps of space; in higher cortex, mixed selectivity expands representational capacity while recurrent structure constrains activity into interpretable trajectories [26]. These converging examples indicate that behavior emerges not from neurons alone, but from a biologically integrated system spanning glia, vasculature, morphology, circuits, and population dynamics.

## 8. Energetic Cost of Cerebral Activity

Neural activity is metabolically demanding. The brain accounts for roughly 20% of the body’s resting energy expenditure despite constituting only 2% of body mass. Most of this energy supports the electrochemical gradients required for action potentials, synaptic transmission, and sustained activity across large neuronal populations, even when no overt behavior occurs [49,50,51]. A substantial fraction is devoted to movement-irrelevant activity in cortical, subcortical, and cerebellar networks. This activity continuously maintains internal representations of plans, predictions, and goals that are essential for adaptive behavior. This persistent, internally generated activity enables the evaluation of alternatives, suppression of irrelevant actions, and preparation of context-appropriate responses before motor output. Thus, preparatory trajectories in the motor cortex’s null space and sustained delay-period activity in prefrontal regions require ongoing synaptic and metabolic support. Although such dynamics do not directly contract muscles, they sustain the cognitive architecture that allows organisms to act flexibly and prospectively under uncertainty. The high baseline cost of maintaining these states reflects their biological indispensability; evolution would be unlikely to tolerate such expense unless it conferred clear adaptive value. Indeed, cerebral energy consumption persists during anesthesia or without sensory stimulation, indicating continuous internal modeling, signal integration, and control maintenance [49,50].

Metabolic constraints are thought to have shaped neural coding toward efficiency. Strategies such as sparse coding, predictive processing, and low-dimensional population dynamics reduce redundancy and spiking frequency while preserving flexibility and the capacity for rapid motor execution [50]. Although neurons account for most cerebral energy use, astrocytes and the vascular system are integral to regulating its delivery.

Astrocytes manage glucose and oxygen supply, coordinate metabolic coupling with neurons, and recycle neurotransmitters, ensuring that resources reach active circuits during high cognitive or motor demand. The vascular system complements this regulation through neurovascular coupling, matching local blood flow to neuronal activity and sustaining both fast signaling and slow integrative processes. The energetic investment in cerebral activity also reflects the need to maintain low entropy against thermodynamic decay. Organisms ‘channel’ this expenditure toward behaviors that secure energy for growth, reproduction, and survival [22]. Adaptive strategies often reduce computational load through body–brain integration, as in marmoset vocal development where lung growth modifies feedback to central pattern generators, eliminating inappropriate calls without neural structural changes. Such mechanisms illustrate how energy-intensive neural operations align with life-sustaining goals, favoring efficient resource use over exhaustive processing [22].

## 9. Behavior Needs Neuroscience (And Vice Versa)

Behavior and neuroscience provide complementary perspectives on a single phenomenon. Behavioral science emphasizes environmental contingencies and observable responses, while neuroscience examines the physiology and organization of the nervous system. Advances in both fields show that neither perspective is sufficient in isolation: neural activity organizes, constrains, and generates behavior, while behavior provides the context through which neural dynamics acquire meaning. Neural data collected under impoverished conditions often fail to generalize to natural cognition, and behavioral models that ignore neural implementation lack explanatory depth [9].

Behavior emerges from dynamically reconfigurable neural networks spanning cortical, subcortical, cerebellar, and neuromodulatory systems. These circuits integrate internal states with environmental context to produce flexible responses [52]. Canonical behavioral processes such as generalization, inhibition, sequence learning, and evidence accumulation are supported by specific mechanisms. Dopaminergic neurons are thought to encode prediction errors, the cerebellum calibrates motor gain and timing, and cortico-thalamic loops sustain attention and working memory. Structured neural dynamics encode internal states, such as belief states, expected rewards, and confidence estimates, shaping action even in the absence of overt movement [1,35].

Modern methods reinforce this integrative view. Dimensionality reduction and manifold learning reveal that motor and cognitive behaviors unfold as low-dimensional trajectories shaped by circuit architecture and experience [16,17]. Reinforcement learning, probabilistic inference, and dynamical systems approaches are increasingly combined with neural recordings to map how latent variables guide both population activity and action. High-throughput behavioral quantification, unsupervised identification of “behavioral motifs,” and targeted perturbations link neural states to multi-scale structure [10,53].

Ethologically grounded neuroscience also strengthens these links. In freely moving rodents, navigation, foraging, prey capture, and social interaction can be decomposed into stereotyped action units mapped to distributed circuits including retrosplenial cortex, striatum, septum, and midbrain [11]. Foraging follows rules of gathering and weighing evidence from the environment, while hunting and tracking in animals without foveas reveal eye-movement strategies that differ from those seen in primates. Social behaviors like kin recognition and dominance are linked to specific brain circuits, such as the lateral septum, which is organized to reflect family relationships (‘nepotopy’).

The relationship between behavior and neuroscience is reciprocal. Ethology and behavioral ecology gain mechanistic grounding from neuroscience, while neuroscience gains ecological validity from behavioral frameworks. Neural perturbations in species-typical traits, such as flexible foraging in rodents, reveal how circuits implement ‘behavioral grammar’. Computational models that incorporate neural constraints improve predictions of ecological decision-making [11]. Collaboration with computer science has accelerated computational ethology, where large-scale datasets and machine learning uncover neurobehavioral principles across species, from gene–behavior associations to whole-brain dynamics [10,54].

A full account of behavior also requires conceptual clarity. Dualistic thinking and the mereological fallacy risk misattributing psychological predicates to isolated brain regions, as when the “amygdala is said to fear.” Psychological functions such as remembering or perceiving are properties of the organism, enacted through the integrated activity of the brain, body, and environment. Isolated regions participate in these functions but do not ‘contain’ them in a literal sense. Avoiding this error requires framing results in terms of neural correlates of behavior, rather than reifying brain structures as agents.

Behavioral science grounds explanations in observable action shaped by contingencies, neuroscience reveals the mechanisms that implement these processes, and computational models provide the formal structures linking inputs, states, and outputs. Integrated together, these perspectives avoid the extremes of reductive localizationism and purely descriptive accounts, offering a biologically grounded framework for adaptive action. In sum, behavior and neuroscience form a single explanatory system. Neural data divorced from behavior risk sterile interpretation, while behavioral theories detached from neural implementation lack mechanistic grounding. A unified science of behavior requires ecological and embodied approaches, recognizing that perception and action exist in reciprocal loops and that brains, bodies, and environments co-constitute adaptive behavior [22].

## 10. Toward a Mechanistic Behavioral Neuroscience

Studying behavior as the output of structured neural dynamics, constrained by metabolic and glial factors, and embedded in closed feedback loops with the environment calls for scientists fluent across levels of analysis, from cellular mechanisms to cognitive processes, and from experimental contingencies to population coding. This interdisciplinary profile is already emerging: researchers increasingly combine behavioral theory, systems neuroscience, computational modeling, and experimental design. They operate at the intersection of disciplines, modeling behavior in terms of latent states, decision policies, and motor hierarchies, and mapping these constructs onto neural population dynamics and circuit-level mechanisms.

Building this kind of expertise requires deliberate educational design rather than passive disciplinary drift. Training programs should integrate neuroscience, biology, physics, mathematics, and computational science, grounding students in theory while equipping them to analyze high-dimensional, naturalistic behavior with modern recording and perturbation methods. Mentorship should reward conceptual synthesis and prepare scientists who resist reducing behavior to a neural proxy or treating neural activity as a cognitive label. Instead, we propose to approach behavior and neural dynamics as inseparable components of a single system: behavior constrains the interpretation of neural signals, while systems neuroscience explains the mechanisms that organize behavior. Computational thinking is central, turning behavioral hypotheses into testable neural predictions while remaining critical of models that oversimplify natural contexts.

Mechanistic behavioral neuroscience advances by uniting ethological observation with systems-level intervention in naturalistic paradigms. Miniaturized implants, closed-loop optogenetics, and unsupervised pose tracking enable perturbations during freely moving behaviors, such as pulsing dopamine during specific behavioral syllables to uncover learning rules. Scaling these methods to complex, dynamic environments and integrating multi-scale models, from sub-second action motifs to evolutionary fitness, will allow testing of Tinbergen’s four questions within a unified, open-science framework [11]. Computational tools now make high-throughput, quantitative ethology feasible. Automated pose estimation, optical flow analysis, and supervised or unsupervised action classification generate standardized behavioral vocabularies across laboratories and species [10]. These methods expose fine-grained effects of neural perturbations, such as optogenetic activation altering courtship frequency, and identify postural primitives that may map onto neural modules. Challenges inherent to naturalistic contexts, including occlusions and environmental variability, are being addressed through multi-sensor integration, for example, by combining RFID with video tracking, enabling precise links between neural manipulations and behavioral structure.

Equally important is maintaining conceptual clarity. Mechanistic explanations must connect neural activity to behavior without collapsing into either reductionism or vague abstraction. Avoiding what has been called “Frankenstein errors”, dismantling systems without reconstructing their functional wholes, requires experiments that preserve ecological relevance, interpret variability as adaptive rather than as noise, and situate findings within developmental and evolutionary trajectories [22]. This broader framework ensures that computational and mechanistic advances remain anchored in the organism’s lived context.

Plenty of empirical work illustrates how this integration operates in practice. For example, hippocampal firing patterns reflect prospective trajectories toward goals, with population sequences predicting choices before execution [29]. Motor cortex preparatory activity represents initial conditions of a dynamical system that evolves to produce movement, revealing low-dimensional population structures governing control [23]. Optogenetic suppression of Purkinje cells modulates movement kinematics with millisecond precision, linking cellular-level inhibition to precise motor outcomes [21]. Stable neural modes in M1 predict electromyographic patterns across tasks, showing how low-dimensional trajectories simplify motor control while retaining flexibility [17]. Ring-like organization in head-direction circuits and toroidal geometries in grid cells demonstrate that geometric population structures can emerge from specific connectivity motifs, testable through perturbation [26].

## 11. Conclusions

Behavior is the observable expression of an organism’s internal dynamics, its predictions, learning, memory, motivations, and intentions, realized within the energy-demanding biology of the nervous system. It is structured, context-sensitive, and expressed across multiple timescales. Explaining behavior without reference to neural mechanisms remains superficial, while interpreting neural signals without behavioral context strips them of meaning. Both perspectives are incomplete in isolation.

Advances in circuit mapping, neural population dynamics, computational modeling, and quantitative behavioral paradigms reveal that behavior and neural activity are inseparable aspects of the same system. A comprehensive science of biological agency requires cultivating researchers fluent in both behavioral theory and neural mechanisms, and recognizing that rigor lies in integration, not reduction. This integration must also be conceptually clear: psychological predicates such as planning or deciding apply to organisms, not isolated brain regions. Neural activity enables behavior but does not ‘contain’ cognition; intentions and latent states are inferred from the interplay of neural dynamics, embodied action, and environmental contingencies. This perspective aligns with embodied and enactive accounts of cognition, situating mind and behavior within closed loops between brain, body, and environment.

This integrative perspective has practical value in both research and clinical settings. In the lab, combining behavioral analysis with neural population dynamics provides reproducible metrics and improves cross-study comparisons [10,53]. In medicine, detailed behavioral profiling linked to neural manifolds can help detect early signs of conditions such as Parkinson’s disease or autism [20,55]. Computational models based on reinforcement learning or predictive processing can guide rehabilitation by predicting how circuits reorganize during recovery [35,37]. Beyond the clinic, recognizing that brains, bodies, and environments jointly shape behavior has implications for education, public health, and technology design, encouraging interventions that are more effective and ecologically grounded [22,52]. Moreover, the tools for an integrative science of behavior already exist. For example, in freely moving rodents, naturalistic foraging can be studied in enriched arenas with high-resolution 3D tracking, unsupervised identification of behavioral “syllables”, and simultaneous neural recordings from hippocampus, striatum, and prefrontal cortex. Closed-loop perturbations allow causal testing, while computational models formalize hypotheses about decision rules and latent states. This type of experiment, already feasible with miniaturized implants, closed-loop optogenetics, and modern pose-tracking, demonstrates that integrative approaches are not aspirational but practical. They exemplify how mechanistic, functional, environmental, and computational dimensions can be studied together.

Integrative frameworks also hold promise for clinical neuroscience. Linking behavioral signatures with neural dynamics could improve diagnosis in movement disorders, autism spectrum conditions, or psychiatric illnesses. For example, microstructural analyses of spontaneous behavior, already informative in rodent models of cerebellar and striatal dysfunction, could provide sensitive diagnostic markers in humans. Combining these markers with neural manifold analysis may uncover circuit-level dysfunctions underlying executive deficits in ADHD, altered planning in schizophrenia, or impaired motor control in Parkinson’s disease. Therapeutically, a mechanistic understanding of how behavior and neural dynamics co-evolve enables targeted interventions. Model-based approaches could guide neuromodulation or rehabilitation strategies by predicting how altered trajectories in neural state space impair planning or decision-making. Importantly, this perspective emphasizes embodiment: interventions should combine pharmacological or neuromodulatory treatments with behavioral training, physical therapy, or environmental modification. Considering glial, vascular, and metabolic contributions broadens the diagnostic and therapeutic scope, supporting treatments that respect the complexity of the whole organism in context.

The future of behavioral neuroscience belongs to researchers who move fluently across levels of analysis, bridging theory and experiment with empirical rigor. By treating behavior and neural activity as co-constitutive, the field can deliver a mechanistically grounded understanding of adaptive action. This integration is not a methodological preference but a biological necessity for explaining how brains, bodies, and environments together generate the richness and flexibility of behavior. Achieving this vision will require not only broad reading and conceptual synthesis but also good old-fashioned hard, sustained work in the laboratory. Progress will not come from sporadic visits to the lab or casual observation, but from long hours of experimentation, analysis, and reflection. Great ideas will not emerge from passive observation or casual distraction; they will be earned through the effort of a generation of scientists who are doers, who read deeply, think critically, and work tirelessly at the lab bench to turn ideas into discoveries.

## Data Availability

No new data were created or analyzed in this study. Data sharing is not applicable to this article.

## Abbreviations

CPG	Central Pattern Generator
GPFA	Gaussian Process Factor Analysis
jPCA	(Joint) Principal Component Analysis
M1	Primary Motor Cortex
PMd	Premotor Cortex
RFID	Radio-Frequency Identification
SLDS	Switching Linear Dynamical Systems
